# Characteristics of cervical disease among symptomatic women with histopathological sample at Hawassa University referral hospital, Southern Ethiopia

**DOI:** 10.1186/s12905-017-0444-5

**Published:** 2017-09-29

**Authors:** Gemechu Ameya, Fekade Yerakly

**Affiliations:** 1 Department of Medical Laboratory Science, College of Medicine and Health Sciences, Arba Minch University, P.O. Box: 21, Arba Minch, Ethiopia; 2 Department of Pathology, School of medicine, College of medicine and health sciences, Hawassa University, Hawassa, Ethiopia

**Keywords:** Cervical cancer, Precancerous lesion, Histopathologic examination, Southern Ethiopia

## Abstract

**Background:**

Cervical cancer is one of the most common cancers of women in developing countries. It is also eminent cause of mortality and morbidity in women worldwide. Symptoms usually develop when the cancer has become invasive and invade nearby tissue. This has significant effect on treatment of cases in area where there is limited awareness. The aim of this study is to describe cervical disease among symptomatic women with a histhological sample at Hawassa University referral hospital, southern Ethiopia.

**Methods:**

Five years retrospective histopathological characteristics of symptomatic cervical cases were studied from January, 2010 to January, 2015 at Hawassa University referral hospital pathology laboratory. Clinical diagnosis of patients, patient’s age, biopsy sample size and type, and microscopic finding of the cases were collected for this study. The data were entered by Epi-Info statistical software version 7 and later exported to SPSS version 20 for analysis. Descriptive analyses such as frequency, mean, and standard deviation were computed.

**Results:**

A total of 513 patients with cervical lesion were examined histopathologically in the study period. The age ranges of the patients were from 17 to 85 years with mean and standard deviation of 42 ± 11 years. Of these symptomatic examined cases, 253 (49.3%) of them were cancerous lesion while non cancerous and precancerous cases account 166 (32.4%) and 51 (9.9%) respectively. Cervical squamous cell carcinoma was the predominant type cancer which accounts 211(83.4%) of all cancerous cervical cases. The proportion of cervical cancer was higher in age group older than 60. The magnitude of cervical cancer and precancerous cases were steadily increasing throughout study periods whereas non cancerous cases were fluctuating.

**Conclusions:**

Cervical cases were associated with wide range of disorders. Cervical cancer was the predominant cervical disease in symptomatic southern Ethiopian women. The high proportion of cervical cancer was observed in post-menopausal age when compared with other cervical cases. Effective uses of low-tech and inexpensive screening tools that already exist and giving awareness about the disease in combination with vaccine could reduce this high magnitude of cervical cancer in study area.

## Background

Cervical disease may range from the simple cervicitis to life treating cervical cancers. Cervical cancer is the most frequently occurring type of reproductive age cancers in women worldwide [1]. According to GLOBOCAN 2012 report, an estimated 14.1 million new cancer cases and 8.2 million cancer-related deaths occurred in 2012. Among these with 528,000 new cases every year, cervical cancer is the fourth most common cancer which is affecting women worldwide [2]. The burden of cervical cancer is quite low in the developed countries like North American with only 6.6 per 100,000 women new cervical cancer cases annually [2]. In contrast, it is most notable and cause of cancer death in the lower-resource countries of sub-Saharan Africa [1]. Almost 70% of the global burden of cervical cancer falls in areas with lower levels of development [3]. In sub-Saharan Africa, about 34.8 new cases of cervical cancer case diagnosed per 100,000 women annually with 22.5 per 100,000 women die from the disease [1].

Similar to other east African countries, in Ethiopia cervical cancer is the most frequent cancer among women and it detected as early as 15 years of age [4]. According to resent WHO report, about 29.43 million women are at risk for cervical cancer. Annual cervical cancer cases were 7095 while 4732 deaths were observed due to cervical cancer. Crude incidence rate of cervical cancer was 16.3 per 100,000 populations [5]. This major difference with developed country may be due to lack of access to effective screening programs and to services that facilitate early detection and treatment as well as low level of awareness about the disease. This can have devastating effects with a very high social, human, and economic cost which affect women in their prime [6].

Cervical cancer arises from the cervix due to the abnormal growth of cells that have the ability to invade or spread to other parts of the body [6]. Histopathologic examination is the accurate method for diagnosis of different cervical lesions [7]. Carcinomas of the cervix can be categorized based on histological morphology into squamous cell carcinomas which is the predominant one, adenocarcinomas, adenosquamous carcinomas, and others [8]. Squamous cell cervical carcinoma tumors are also graded based on their degree of differentiation into well-differentiated, moderately-differentiated, and poorly-differentiated or undifferentiated [9].

Human papillomavirus (HPV) infections are the most common cause of cervical cancer through worldwide [4, 10]. Among more than 100 different types, infection with HPV 16 and 18 has been associated with more than 70% of cervical cancers [11]. Their type has not been associated either with survival or with morphological types of cancer. The virus can cause persistent infection and it will be risk for progression to precancerous lesion and cancer of the cervix [4, 10]. Persistent HPV infection seems to be able to lead to preinvasive cancer (CIN III or cancer in situ) on its own, but cofactors are also required for development of invasive cancer [12].

Limited data are available on the HPV burden in the general population of Ethiopia. However, study conducted in Gondar, northwest Ethiopia indicates that about 92.6% of cervical cancer sample showed HPV positive. Among these, 76% of them are HPV type 16 [13]. According to WHO human papillomavirus and related diseases, prevalence of HPV was 97%, 76.9% and 26.5% among cervical cancer cases, high-grade and low-grade precancerous lesions respectively. The predominant types of human papillomavirus in Ethiopia were HPV 16 and HPV 18 [5].

The study carried out in different part of Ethiopia showed that knowledge about cervical cancer is very low. In study that is conducted on northwest Ethiopia, only 31% of women were knowledgeable about cervical cancer [14]. From the findings of the studies that are done in Addis Ababa and southwest Ethiopia the awareness of people was very low. According to their thought, cervical cancer cause as a result of undertaking unacceptable behaviors or violating of social taboo [15]. Due to these reason patients seek medical care in advanced stage of cervical cancer which became difficult to treat.

According to recent WHO report, in Ethiopia national cervical cancer screening program is not established and screening coverage was only 0.8% [5]. In area where this research was done, the number of screening test conducted was very few, and histopathologic diagnosis was carried out for patient with symptom. We conduct this study on the formol saline fixed cervical biopsy sample taken at Hawassa University referral hospital and other its district hospitals. A routine hematoxylin and eosin staining technique was used to report histopathologic diagnosis. In this study area, so far there is no study conducted on histopathologic examination of cervical sample. Therefore, the aim of this study is to characterize cervical disease among symptomatic women with a histhological sample at Hawassa University referral hospital, southern Ethiopia.

## Methods

Retrospective study was conducted on all cervical biopsy samples that were taken from symptomatic women of Hawassa district, southern Ethiopia from January, 2010 to January, 2015. Hawassa is the capital city of Southern Nations, Nationalities, and Peoples’ region of Ethiopia. This region has a total population of 15,321,000 and nearly half of them were females according to the 2007 census report [16]. In this study area, cervical biopsy were taken and fixed with 10% formol saline fixative transported to Hawassa University referral hospital pathology laboratory for diagnosis. Upon receipt of all biopsy samples, identification number was given for each patient and the diagnosed results were recorded in both hardcopy and softcopy.

After the biopsy sample reached histopathology laboratory, a histopathology technicians give new laboratory number and ensure as fixative fluid was enough. Then senior pathologists examine the gross anatomy of the samples and take representative bits of the biopsy from the samples. The histopathology technicians of the laboratory processed these bits of tissues by the help of Leica TP 1020 tissue processor machine. During tissue processing, water was removed by using a number of increasing concentration of ethanol. This was followed by a hydrophobic clearing agent xylene to remove the alcohol and finally molten paraffin wax was used to infiltrate and replace the xylene. After that, the tissue was embedded by the help of Leica EG 1160 tissue embedding instrument so that the tissue in paraffin wax is firmly attached to tissue cassette. Rotary microtome was used to cut 3-5 μm section. Then the section was floated on the surface of water bath adjust at temperature just below the melting point of paraffin wax. This help to pick section on microscopic slide and to remove wrinkles appeared during sectioning. After the sectioned tissue was picked on microscopic slide, it was placed in a warm oven adjusted at 60 °C for about 30 min to so that it adheres to the slide. Then all cervical biopsy samples were stained with haematoxylin and eosin according to prepared standard operation procedure. Finally, the microscopic examination of stained slides was examined and reported by senior pathologist. Histopathologic classification of cervical cancer cases were also carried out according to the world health organization classification of cervical tumors [17].

For this study, we collected the information such as date and year of examination, clinical diagnosis of patient, patient’s age, type of biopsy samples, biopsy sample size and site, and microscopic result of the samples. The gathered data was checked and entered by Epi-Info statistical software version 7 and later exported to SPSS version 20 for analysis. Descriptive analyses such as frequency, mean, and standard deviation were used.

## Results

In the 5 years period (from January 2010 to January 2015), a total of 513 cervical biopsy samples were received from Hawassa University referral hospital pathology laboratory. The age range of patients was from 17 to 85 years with mean and standard deviation of 42 ± 11 SD. About 39% of women with histopathologic sample were age group between 31 and 40 years old. Majority of samples 391 (76.2%) taken in study period were punch biopsy.

Of 513 cervical biopsy examined in study period, only 43 (8.4%) of them were reported as normal while the majority of them showed the same pathologic problems which range from simple cervicitis to cervical cancer. From all cervical cases, 253 (49.3%) of symptomatic women were diagnosed for cervical cancer. Precancerous and non-cancerous cervical cases account 51 (9.9%) and 166 (32.4%) respectively (Table 1).

**Table 1:** Numbers and percentages of cervical lesion in symptomatic women attending Hawassa University referral hospital (*n* = 513)

Type of Cervical Lesion	Case number	In percent (%)
Precancerous cervical lesion	51	9.9
Cancerous cervical lesion	253	49.3
Non-cancerous cervical lesion	166	32.4
Normal cervical biopsy	43	8.4
Total	513	100

About 80% of cervical cancer cases of the symptomatic women were observed in age above 60 years old while the least proportion was observed in age group less than 30 years old. In contrast, high proportion of non cancerous cases was observed in age group less than 30, and low proportion was exhibited in older age. On the other hand, there was a little variation in proportion of precancerous cases between age groups, but its highest proportion was observed in age range between 41 and 50 years which accounts about 12% of cervical cases in the age group **(**Table 2 **)**.

**Table 2:** Pattern of cervical lesion in symptomatic women attending Hawassa University referral hospital by age group (n = 513)

Age of Patients	Precancerous cervical lesion	Cancerous cervical lesion	Non-cancerous cervical lesion	Normal cervical biopsy	Total (%)
<30	6 (8.3%)	24 (33.3%)	36 (50%)	6 (8.3%)	72 (100%)
31-40	19 (9.4%)	84 (41.8%)	77 (38.3%)	21 (10.4%)	201 (100%)
41-50	17 (11.9%)	74 (52.1%)	38 (26.8%)	13 (9.2%)	142 (100%)
51-60	7 (9.6%)	51 (69.9%)	13 (17.8%)	2 (2.7%)	73 (100%)
>60	2 (8%)	20 (80%)	2 (8%)	1 (4%)	25 (100%)
Total	51 (9.9%)	253 (49.3%)	166 (32.4%)	43 (8.4%)	513 (100%)

In current study, squamous cell carcinoma (SCC) also accounts about 211 (83.4%) of total cervical carcinoma is followed by adenocarcinoma which accounts 39 (15.4%) and the others are adenosquamous carcinomas, and mucinous adenocarcinoma. Based on degree of differentiation, histological grading of SCC was also determined according to WHO grading, and moderately differentiated SCC was predominant one in study period. There was no great difference between proportions of grading of SCC; however; moderately differentiated SCC was high in all age groups (Table 3).

**Table 3:** Distribution of cervical squamous cell carcinoma by histological grading and age group in symptomatic women attending Hawassa University referral hospital (*n* = 211)

Age of patient	Histological grading of SCC			
	Poorly-differentiated	Moderately-differentiated	Well-differentiated	Total (%)
<30	6 (35.3%)	9 (52.9%)	2 (11.8%)	17 (100%)
31-40	11 (15.3%)	33 (45.8%)	28 (38.9%)	72 (100%)
41-50	12 (19.4%)	28 (45.2%)	22 (35.5%)	62 (100%)
51-60	9 (21.9%)	19 (46.3%)	13 (31.7%)	41 (100%)
>60	7 (36.8%)	9 (47.4%)	3 (15.8%)	19 (100%)
Total	45 (21.3%)	98 (46.4%)	68 (32.2%)	211 (100%)

Chronic non-specific cervicitis which accounts 53.6% of non-cancerous cervical diseases was the second cause of cervical lesion. In the study period, Endocervical polyp was the second cause of non-cancerous cervical diseases. Furthermore, among precancerous cervical lesion, high grade cervical intraepithelial neoplasia was the predominant one.

**Fig. 1:**
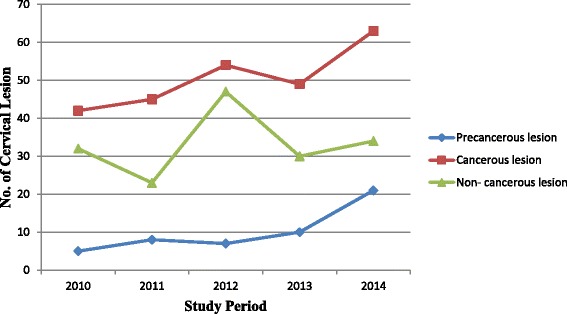
The 5 years trend of cervical lesion in symptomatic women attending Hawassa University referral hospital

Though out the study years, cervical cancer was the major cause of cervical disease in symptomatic women who were attending Hawassa University referral hospital. Its magnitude was also almost increasing in all study years except in 2013. In 2010, the magnitude of cervical cancer seems minimal but there was no great difference of proportion with other cervical disease in the study periods. Similarly, the trend of precancerous cervical lesion was increasing though out the study periods. On the other hand, a 5 year trend of non-cancerous cervical lesion was fluctuating in the study years. In 2012, the magnitude of non-cancerous cases was high whereas the lowest proportion with other cervical diseases was observed in 2014 (Fig. 1).

## Discussion

Nowadays, more than twice as many people die from cancer than from infectious diseases. New cancer cases and deaths are also more common in low and middle-income countries. Cervical cancer is the second killer cancer of women next to breast cancer. In our study, it is also the predominant cervical diseases which account 49.32% in symptomatic women. Cervical cancers do not formed suddenly but the normal cervical cells gradually develop pre-cancerous changes that turn into cancer. Current study shows that about 10% of cases were precancerous change. This indicates that the magnitude of precancerous cases were by far less than cancerous cases. The possible reason for this discrepancy may be due to less awareness of cervical cancer screening in the area and women also visit the hospital after symptom is observed [14, 15].

In our study, the magnitude of cervical cancer in postmenopausal (age group above 50 years old) women was about 72% of cervical disease in symptomatic women. This finding was higher than the finding of the study conducted in Addis Ababa that was 51.6% of postmenopausal bleeding of patient [18]. The differences may be due to the restricted samples to those from women who have postmenopausal bleeding. Furthermore, the observed difference may be due to the difference in socio-demographic status of the patient and difference in the level of awareness between study populations.

In this study, SCC was the predominant type of cancer which accounts about 83% of cervical cancer. Unlike our finding, study conducted in Malaysia showed that SCC was the second cervical lesion with 29% of cervical lesion next to precancerous cervical lesion which account 42% of cervical lesion [19]. The observed difference may be because of little awareness and screening in Ethiopia and most lesions are already cancerous when diagnosed whereas in other places like Malaysia, where screening is undertaken, this was not the case [19].

Adenocarcinoma encountered about 15% of the cancerous cases of symptomatic women in current study. Some authors showed that adenocarcinoma was associated with a worse prognosis when compared to SCC [20]. Magnitude of precancerous cervical lesion and poorly-differentiated SCC were less than moderately differentiated and well differentiated SCC. According to study conducted at the University of Michigan, patients with a well-differentiated tumor had more survival rate (Survival rate 85%) than those with a poorly differentiated tumor (Survival rate 57%) [21]. Other major determinant for the prognosis of cervical cancer is the stage at which the patient present. The study also shows that most patients in developing countries including Ethiopia present late with advanced stage disease [22]. This markedly diminished chance of success of treatment and study also indicates that there is high death rate in later stage of cervical cancer [5, 23]. In this study, histological classification of SCC was made according to WHO histological grading. The histological differentiation stage is strongly associated with tumor behavior. Usually poorly-differentiated is more aggressive than the well differentiated counterpart [24, 25]. The histological classification is intended to facilitate the comparison of results and outcomes. It is also useful to pathologists, gynecologist, radiotherapists, oncologists, and epidemiologists in classification and characterization of cancer. Again, it is important for a reliable prognosis to be established and it provides the basis for clinical management of patients. As observed in our study, majority of malignant lesion of the cervix were squamous cell carcinomas, but other tumor types must be distinguished pathologically because different phenotypes have different biological behavior [8, 9].

In this study, the minimum age of the patient in which cervical cancer was reported was 17 years old. In the other study which is conducted in Ethiopia, cervical cancer was detected in 15 years old [4]. As a whole, the proportion of cervical cancer increases with age of the patient whereas non-cancerous cervical lesion was inversely proportional with age. In developing countries for example, Ethiopia where there is no well-organized screening program, this pattern is expected and the only way to reverse that is by investing in screening and awareness. Study also shows that one-third of cancer deaths can be prevented through screening tests, vaccinations, and lifestyle changes [26].

In this study, the magnitude of precancerous cervical cancerous lesion was 10% which is comparable with study done in Nigeria (6%) [27]. High prevalence of HPV and human immunodeficiency virus (HIV) in sub-Saharan countries can be contributor for the high rate of precancerous and cancerous cervical cases. In the study which is done in southern Ethiopia, HIV positive women, high prevalence (22.1%) of precancerous cervical lesion was reported. The observed difference with our study is due to difference in study population. In their study, the patients are immunocompromised and they are susceptible for different infection disease including HPV. The other possible reason is most of precancerous lesion is asymptomatic and patient might not seek screening service unless they experience some sort of sign and symptom which is common in late stage of cancer in our case [14, 15].

In this study, the second predominant cervical disease was non-cancerous lesion; among them 53.6% of them was chronic nonspecific cervicitis. Similar study that is conducted in Nigeria, chronic nonspecific cervicitis was the most commonly encountered lesion constituting 72.2% of cervical inflammatory lesions [28]. Similarly, result that is observed in Puducherry, India was high which accounts 89.23% of cervicitis [29]. Cervicitis can be caused by different infectious pathogens which are transmitted by sexual intercourse. In this study, histopathological differential diagnosis of cervicitis was not performed. However, the study showed that the majority of cervicitis was caused by *C. trachomatis*, *N. gonorrhoeae* and herpes simplex virus [30]*.* In current study, the magnitude of endocervical polyp was 39.8% of non-cancerous lesion, and it is about 13% of total cervical lesion which was higher than study conducted in Danish (7.8%) [31].

Magnitude of cervical cancer was almost increasing in study periods. This may be due to increment in the level of awareness of women. Nowadays, as compared to previous time in Ethiopia, ministry of health also gives a great attention for this killer disease; as a result, women are initiated for screening. This may also the reason why the trend of precancerous cervical lesion was increasing throughout the study period. Non-cancerous cervical lesion was fluctuating in the study years and high magnitude was observed in 2012. The possible reason for this increment may be lack of well organized screening and control scheme in the study area, and women mainly screened in case experience symptom. In general, cervical cancer is remaining major health problem of southern Ethiopian women.

As limitation, our study is retrospective and based on histopatholagical sample obtained in pathology laboratory. Screening test of asymptomatic cases was very limited in study area and for this reason we consider only symptomatic cases. There was no data of histopathologic differentiation for adenocarcinoma. The way women get better awareness on the screening of cervical cancer should be searched so that women in study area can know their status before a lesion advanced.

## Conclusion

Cervical diseases can be associated with wide range of disorders. It was the predominant cervical disease in symptomatic women who were attending Hawassa University referral hospital. In our study, in contrast to non-cancerous cervical disease, the proportion of cervical cancer was high in older age. The magnitude of cervical precancerous changed and cervical cancer was increasing throughout study periods whereas non-cancerous cervical disease was fluctuating. Effective using of low-tech and inexpensive screening tools that already exist and giving awareness about the disease with vaccination could reduce this high magnitude of cervical cancer in study area.

## Abbreviations

CIN: Cervical intraepithelial neoplasia
HIV: Human immunodeficiency virus
HPV: Human papillomavirus
SCC: Squamous cell carcinoma
